# Effect of extending the oral administration period of 5‐aminolevulinic acid on diagnostic accuracy and treatment outcomes for non–muscle‐invasive bladder cancer

**DOI:** 10.1002/bco2.70173

**Published:** 2026-02-12

**Authors:** Hideo Fukuhara, Ryu Shigehisa, Shinkuro Yamamoto, Satoshi Fukata, Kenta Saito, Yasuhiko Shibanaka, Keiji Inoue

**Affiliations:** ^1^ Department of Urology and Center for Photodynamic Medicine Kochi Medical School Nankoku Japan; ^2^ SBI Pharmaceuticals Co., Ltd Tokyo Japan

**Keywords:** 5‐aminolevulinic acid, non–muscle‐invasive bladder cancer, photodynamic diagnosis, transurethral resection of bladder tumour, white light

## Abstract

**Objectives:**

To evaluate the treatment outcomes of 5‐aminolevulinic acid hydrochloride (5‐ALA) photodynamic diagnosis (PDD)‐assisted transurethral resection of bladder tumour (TURBT) (ALA PDD‐TURBT, hereinafter referred to as ‘ALA‐PDD’) exceeding 4 h after ALA administration for non–muscle‐invasive bladder cancer (NMIBC).

**Patients and methods:**

This retrospective single‐centre study included 386 patients who had undergone TURBT with or without 5‐ALA for NMIBC between January 2018 and December 2024. Patients who received 5‐ALA were divided into two groups based on 5‐ALA exposure times before TURBT: 2–4 h and 4–8 h groups. The diagnostic sensitivity and specificity of procedures performed after the two exposure times were calculated by comparing cystoscopy findings with pathological findings in the ALA‐PDD group. Recurrence‐free survival (RFS) and progression‐free survival (PFS) rates of NMIBC patients in the white‐light (WL) and ALA‐PDD groups were examined using Kaplan–Meier curves.

**Results:**

When the same lesion was evaluated using WL and fluorescent light (FL) modes, the sensitivity was 62.6% for the former and 93.2% for the latter. Furthermore, when the FL mode was divided into two ALA‐PDD groups, the sensitivity was 93.9% in the 2–4 h group and 91.3% in the 4–8 h group (*p* = 0.29). On the other hand, RFS was significantly longer in both the 2–4 h and 4–8 h ALA‐PDD groups than in the WL group (*p* < 0.05), with no significant difference in RFS between the 2–4 h and 4–8 h ALA‐PDD groups (*p* = 0.105).

**Conclusion:**

The clinical efficacy of ALA‐PDD, in terms of sensitivity and recurrence, was maintained even when the 5‐ALA administration time was extended from 2 to 4 h to 2–8 h prior to TURBT.

## 1 INTRODUCTION

Photodynamic diagnosis‐assisted transurethral resection of bladder tumour (PDD‐TURBT) for non–muscle‐invasive bladder cancer (NMIBC) is an important surgical technique for urologists, in terms of improving both diagnostic accuracy and treatment outcomes.^1^, ^2^, ^3^, ^4^, ^5^, ^6^, ^7^ In Japan, the Drug Manufacturing and Marketing Application (DMMA) for visualization of NMIBC during PDD‐TURBT using ALAGLIO® (SBI Pharmaceuticals Co., Ltd., Tokyo, Japan; 5‐aminolevulinic acid hydrochloride) was approved and became covered by health insurance in 2017, making it possible to perform this procedure in clinical practice. In the DMMA, the recommended dosage of 5‐ALA (ALAGLIO®) was administered orally 3 h (range: 2–4 h) prior to cystoscope insertion, and it was not recommended to perform ALA PDD‐TURBT (hereinafter referred to as ‘ALA‐PDD’) beyond 4 h after 5‐ALA administration. However, in the field of urology, scheduling of ALA‐PDD within the 2‐h window after 5‐ALA administration often poses significant practical challenges, due to its impact on the feasibility of concurrently performing robotic surgery, laparoscopic surgery and multiple TURBTs on the same day. We previously investigated the diagnostic accuracy of ALA‐PDD based on real‐world data from 76 patients, including 15 in whom TURBT unintentionally exceeded 4 h, and reported a diagnostic sensitivity of 85.7% in the group treated 2–3 h after oral 5‐ALA administration, 90% in the group treated 3–4 h after the administration and 96.8% in the group treated 4 h or more after ALA administration, with no significant difference between the three groups.^8^

To improve the usability of ALA‐PDD, Taoka et al. conducted a Phase III study (SPP2C102 study) to evaluate the diagnostic accuracy and safety of ALA‐PDD when the time range of the oral administration was extended from 2 to 4 h to 4 to 8 h before cystoscope insertion.^9^ In the full analysis set of a total of 144 patients in this study, ALA‐PDD under fluorescent light (FL) mode demonstrated a sensitivity of 95.3% and specificity of 52.7%, whereas the white light (WL) mode had a sensitivity of 61.1% and specificity of 95.2%. The sensitivity of the extended administration period (4–8 h) was equivalent to that of the conventional period of within 4 h with ALA‐PDD under the FL mode. Based on the results of this Phase III study, the appropriate timing for oral administration of 5‐ALA was officially approved as 2–8 h before cystoscope insertion in September 2024 in Japan.

Although some reports have been published on the diagnostic accuracy of ALA‐PDD exceeding 4 h after 5‐ALA administration, there have been no reports on disease recurrence or progression after the procedure based on real‐world data. Therefore, we focused on the treatment outcomes of ALA‐PDD performed beyond 4 h after 5‐ALA administration.

## 2 PATIENTS AND METHODS

### 2.1 Patients, dosage regimen and technical procedures

This retrospective single‐centre study enrolled 516 patients who had undergone TURBT at Kochi Medical School Hospital between January 2018 and December 2024. The Institutional Review Board of Kochi Medical School Hospital approved the study (approval number 2025‐52) and waived the need for informed consent in accordance with the opt‐out policy of Kochi Medical School Hospital. The study was conducted in accordance with ethical principles for medical research on human subjects, including research on identifiable human materials and data, as stated in the Declaration of Helsinki (64th World Medical Association General Assembly, Fortalenza, Brazil, October 2013).

Patients in whom NMIBC was suspected based on preoperative evaluations with outpatient cystoscopy and urine cytology were designated to be treated with TURBT. The decision to perform ALA‐PDD is made on an individual physician's judgement based on concerns about adverse events (AEs) of 5‐ALA. Patients with a risk factor of 5‐ALA induced hypotension such as chronic heart failure, atrial fibrillation, haemodialysis and severe carotid artery stenosis were allocated to WL mode.

A total of 516 patients who had undergone TURBT were initially included in this analysis of diagnostic accuracy. After excluding patients with no tumour, muscle‐invasive bladder cancer (MIBC) and unknown time of 5‐ALA intake, 161 patients were in the WL group, and 225 patients were in the ALA‐PDD group. After excluding 14 patients with off‐label usage of 5‐ALA with respect to exposure time before TURBT from the ALA‐PDD group, 144 patients were analysed in the 2–4 h group and 67 in the 4–8 h group based on their 5‐ALA exposure time range. The definition of off‐label use was patients that deviated from the new regimen of 5‐ALA (bladder cystoscope insertion 2–8 h prior) approved after September 2024. The dosage regimen and technical procedures followed were as described in a previous report.^3^ Briefly, 5‐ALA was dissolved in 50 ml of water and administered orally at a dose of 20 mg/kg body weight in the ALA‐PDD group, at least 2 h before TURBT. TURBT guided by PDD was performed using an IMAGE 1S OPAL1 PDD system (Karl Storz GmbH &Co. KG, Tuttlingen, Germany) and a D‐LIGHT C/AF (300 w Xenon bulb) equipped with a bandpass filter to transmit blue light (excitation wavelength: 375–445 nm), which is able to detect the red fluorescence generated by 5‐ALA through a telescope (HOPKINS II PDD telescope 4 mm, 30°C) by switching between the WL and the FL modes. After anaesthesia, the same lesions were observed in WL mode and FL mode during ALA‐PDD. Subsequently, random bladder biopsies were performed only in lesion‐free regions among the seven predefined regions: right lateral wall, posterior wall, left lateral wall, trigone, dome/anterior wall, bladder neck, and prostatic urethra. After recording the macroscopic findings, tissue samples were obtained and specimens showing either ‘weak’ or ‘positive’ fluorescence intensity were defined as tumour positive.

### 2.2 Analysis of diagnostic accuracy

As previously reported, the diagnostic accuracy in the ALA‐PDD group was verified by comparing cystoscopy findings with pathological results to determine the sensitivity and specificity of ALA‐PDD for identifying tumours intraoperatively.^10^ In this study, we calculated the diagnostic sensitivity and specificity in the ALA‐PDD group for all lesions, including carcinoma‐in situ (CIS), by comparing cystoscopy findings with pathological findings while switching between the WL and FL modes during TURBT. Based on the 5‐ALA exposure time before TURBT, patients were divided into two groups: the 2–4 h group (2 h or more but less than 4 h), and the 4–8 h group (4 h more but not exceeding 8 h), and the diagnostic accuracy for each exposure time was analysed. For convenience, the 5‐ALA exposure time was defined as the period between the oral administration of 5‐ALA and cystoscope insertion.

### 2.3 Protocol for outpatient follow‐up cystoscopy after TURBT

Whether a second TUR was performed after initial TURBT depended on the judgement of individual physicians, based on pathological results (Ta or T1). The decision to perform a single immediate instillation of pirarubicin after TURBT was also at the discretion of individual physicians. The outpatient follow‐up protocol was as follows: Cystoscopy and urine cytology were performed every 3 months for 2 years, then every 6 months for the next 3 years, and annually thereafter.

Disease progression was defined as recurrent disease presenting as MIBC, positive lymph nodes, or distant metastases in any of these patients. Recurrence was defined broadly to include intravesical tumour recurrence confirmed as urothelial carcinoma (including dysplasia and atypical epithelium) as well as disease progression, as previously described following TURBT.

### 2.4 Postoperative analysis of bladder cancer recurrence and progression to MIBC in the WL and ALA‐PDD groups during follow‐up for NMIBC

RFS and PFS rates within the 1000‐day follow‐up period were examined using Kaplan–Meier curves in the WL and ALA‐PDD groups in NMIBC patients. Furthermore, the frequency of bladder cancer recurrence during the follow‐up period was calculated and examined using the person–time method ([number of bladder recurrences]/[total number of follow‐up days]/10 000 days). The frequency of bladder recurrence after the first TURBT was compared between the WL and ALA‐PDD groups. RFS, PFS and person–time methods were also analysed by time stratum for each 5‐ALA exposure time (2–4 h and 4–8 h).

### 2.5 Statistical analysis

Significant differences were determined using two‐side Student's *t*‐test or Welch's *t*‐test for parametric data. McNemar's test, *χ* ^2^ test and Fisher's exact test were used for nonparametric data. Log‐rank testing was used for comparison of RFS. To examine differences in the distribution of categorical variables among three or more groups, a *χ* ^2^ test was first performed on the overall contingency table. When a significant difference was observed in the overall test, pairwise comparison among groups was conducted using individual *χ* ^2^ tests. To control for Type I errors due to multiple comparisons, the resulting *p* values were adjusted using Holm's method. The same approach was applied to compare RFS and PFS among three or more groups. For these survival outcomes, overall tests were followed by pairwise comparisons, and Holm's method was used to adjust the *p* values. *p* values less than 0.05 were considered statistically significant. Statistical analysis was performed using Microsoft Excel and R version 4.4.2 software.

## 3 RESULTS

### 3.1 Patients' characteristics

**FIGURE 1:**
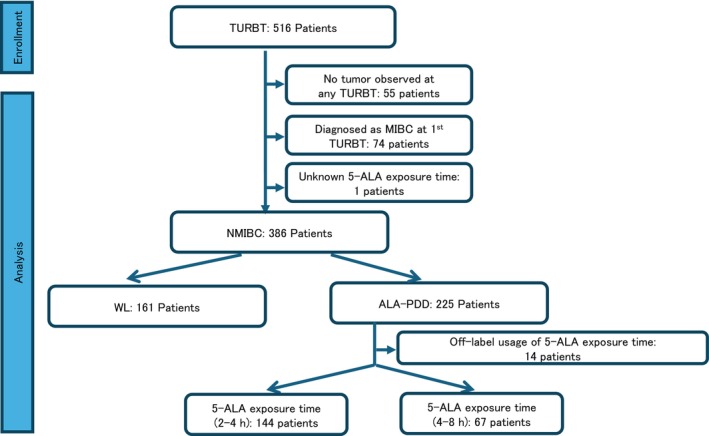
Flow diagram of patient inclusion throughout in study. One hundred sixty‐one patients in the WL group and 225 in the ALA‐PDD group were diagnosed with NMIBC and included in the analysis. Among ALA‐PDD group, 14 patients who received 5‐ALA off‐label exposure times were excluded from the analysis comparing different 5‐ALA exposure time ranges within the ALA‐PDD group. ALA‐PDD, 5‐aminolevulinic acid hydrochloride photodynamic diagnosis; NMIBC, non–muscle‐invasive bladder cancer; WL, white light.

The patients included in this analysis are shown in Figure 1. Of the 386 patients who underwent TURBT for NMIBC, 161 were included in the WL group and 225 in the ALA‐PDD group. After excluding 14 patients with off‐label usage of 5‐ALA with respect to exposure time before TURBT from the ALA‐PDD group, 144 patients were analysed in the 2–4 h group and 67 in the 4–8 h group based on their 5‐ALA exposure time range. Table 1 shows the baseline clinicopathological characteristics of the patients. There were statistically significant differences in age, number of tumours, largest tumour diameter, CIS, pathological stage, residual tumour at first TURBT and postoperative intravesical BCG therapy between the WL and ALA‐PDD groups, and no statistically significant differences in terms of the other clinicopathological characteristics.

**TABLE 1:** Characteristics of patients in this study.

		WL	ALA‐PDD	*p* value	5‐ALA exposure time (h)		*p* value
					2–4	4–8	
Enrolled patient	*N*	161	225		144	67	
Age	Mean ± *SD*	77.0 ± 9.7	72.6 ± 9.2	*p* < 0.05^a^ (0.00001)	71.8 ± 9.4	75.1 ± 8.9	*p* < 0.05^a^ (0.01720)
	Min, median, max	24, 78, 94	34, 73, 89		34, 73, 88	51, 76, 89	
Gender, *N* (%)	Male	118 (73.3)	180 (80.0)	0.12139^b^	117 (81.3)	52 (77.6)	0.53782^b^
	Female	43 (26.7)	45 (20.0)		27 (18.8)	15 (22.4)	
Past history, *N* (%)	Primary	123 (76.4)	162 (72.0)	0.33244^b^	104 (72.2)	49 (73.1)	0.89012^b^
	Recurrence	38 (23.6)	63 (28.0)		40 (27.8)	18 (26.9)	
ECOG performance status, *N* (%)	0	102 (63.4)	153 (68.0)	0.34184^b^	107 (74.3)	37 (55.2)	*p* < 0.05^b^ (0.00558)
	1–3	59 (36.6)	72 (32.0)		37 (25.7)	30 (44.8)	
Number of tumours, *N* (%)	Single	128 (79.5)	104 (46.2)	*p* < 0.05^b^ (0.00000)	71 (49.3)	28 (41.8)	0.30858^b^
	Multiple	33 (20.5)	121 (53.8)		73 (50.7)	39 (58.2)	
Largest tumour diameter, *N* (%)	<3 cm	128 (79.5)	201 (89.3)	*p* < 0.05^b^ (0.00727)	127 (88.2)	64 (95.5)	0.12883^c^
	≥3 cm	33 (20.5)	24 (10.7)		17 (11.8)	3 (4.5)	
CIS, *N* (%)	Absence	143 (88.8)	162 (72.0)	*p* < 0.05^b^ (0.00006)	108 (75.0)	44 (65.7)	0.15989^b^
	Presence	18 (11.2)	63 (28.0)		36 (25.0)	23 (34.3)	
Pathological stage, *N* (%)	pTa	80 (49.7)	100 (44.4)	*p* < 0.05^b^ (0.00066)	69 (47.9)	26 (38.8)	0.27360^c^
	pTis	10 (6.2)	46 (20.4)		31 (21.5)	13 (19.4)	
	pT1	59 (36.6)	71 (31.6)		39 (27.1)	27 (40.3)	
	Others^e^	12 (7.5)	8 (3.6)		5 (3.5)	1 (1.5)	
High‐grade tumour, *N* (%)	No	29 (18.0)	26 (11.6)	0.07354^b^	21 (14.6)	3 (4.5)	*p* < 0.05^c^ (0.03567)
	Yes	132 (82.0)	199 (88.4)		123 (85.4)	64 (95.5)	
Residual tumour at first TURBT, *N* (%)	No	135 (83.9)	219 (97.3)	*p* < 0.05^b^ (0.00000)	139 (96.5)	67 (100)	0.18089^c^
	Yes	26 (16.1)	6 (2.7)		5 (3.5)	0 (0)	
Second TUR, *N* (%)	No	161 (100)	221 (98.2)	0.14377^c^	140 (97.2)	67 (100)	0.30953^c^
	Yes	0 (0)	4 (1.8)		4 (2.8)	0 (0)	
Postoperative intravesical BCG therapy, *N* (%)	No	151 (93.8)	189 (84.0)	*p* < 0.05^b^ (0.00342)	125 (86.8)	52 (77.6)	0.09085^b^
	Yes	10 (6.2)	36 (16.0)		19 (13.2)	15 (22.4)	
Follow‐up days	Mean ± *SD*	577 ± 508	674 ± 497	0.06407^d^	704 ± 556	643 ± 331	0.32681^d^

*Note*: The number in parentheses below the statistical test result indicates the *p* value. Values of *p* < 0.05 were considered statistically significant.

Abbreviations: BCG, Bacillus Calmette‐Guérin; CIS, carcinoma‐in situ; ECOG, Eastern Cooperative Oncology Group; *SD*, standard deviation; TUR, transurethral resection; TURBT, transurethral resection of bladder tumour.

^a^ *p* values were calculated using Student's *t*‐test.

^b^ *p* values were calculated using *χ* ^2^ test.

^c^ *p* values were calculated using Fisher's exact test.

^d^ *p* values were calculated using Welch's *t*‐test.

^e^ Patients with dysplasia or atypical epithelium were included.

### 3.2 Diagnostic accuracy and number of tumour lesions detected under WL mode and FL mode in ALA‐PDD group

The total number of positive and negative lesions in the 225 patients in the ALA‐PDD group was 486 and 900, respectively. To compare diagnostic sensitivity and specificity across the same conditions, patients and lesions, TURBT was performed during ALA‐PDD by switching between WL and FL modes to observe lesions, and finally the results were compared with pathological findings. The diagnostic accuracy was as follows: sensitivity (*p* < 0.05) was 62.6% in the WL mode and 93.2% in the FL mode, and specificity (*p* < 0.05) was 96.4% and 72.4%, respectively, in the two modes (Table 2A). The mean number of tumours detected per patient was 1.35 ± 1.12 in the WL mode and 2.01 ± 1.38 in the FL mode (*p* < 0.05), indicating higher performance under the FL mode (Table 2A). In this study, the false‐positive lesions analysed in the ALA‐PDD group included histologically normal tissue, cystitis, hyperplasia, squamous metaplasia and papilloma. Among these, 98.9% (890 out of 900 specimens) of the false‐positive lesions were histologically normal (data not shown). Of the total of 486 positive lesions from patients in the ALA‐PDD group, 148 were CIS positive lesions. The sensitivity of detecting CIS positive lesions was significantly higher in the FL than the WL mode, at 93.2% and 25.7%, respectively. The mean number of CIS detected per patient (mean ± *SD*) was also significantly higher in the FL than the WL mode, at 0.61 ± 1.23 and 0.17 ± 0.58 (*p* < 0.05), respectively (Table 2B).

**TABLE 2:** Diagnostic accuracy and number of tumours or carcinoma in situ detected under white light (WL) mode and fluorescence light (FL) mode in ALA‐PDD group.

(A)					
Mode	WL(+) or FL(+)/total positive lesion	Sensitivity (%) (95% CI)	WL(−) or FL(−)/total negative lesion	Specificity (%) (95% CI)	Tumours/pts, mean ± *SD*
White light (WL)	304/486	62.6 (58.1–66.8)	868/900	96.4 (95.0–97.5)	1.35 ± 1.12
Fluorescence light (FL)	453/486	93.2 (90.5–95.2)	652/900	72.4 (69.4–75.3)	2.01 ± 1.38
*p* value		*p* < 0.05^a^ (0.00000)		*p* < 0.05^a^ (0.00000)	*p* < 0.05^b^ (0.00000)

(B)			
Mode	WL(+) or FL(+)/CIS positive lesion	Sensitivity (%) (95% CI)	CIS/pts. mean ± *SD*
White light (WL)	38/148	25.7 (19.0–33.6)	0.17 ± 0.58
Fluorescence light (FL)	138/148	93.2 (87.6–96.5)	0.61 ± 1.23
*p* value		*p* < 0.05^a^ (0.00000)	*p* < 0.05^c^ (0.00000)

*Note*: (A) Comparison of the diagnostic accuracy and number of tumours detected between white light (WL) mode and fluorescence (FL) mode in ALA‐PDD group; (B) Comparison of diagnostic accuracy and number of carcinoma in situ detected between WL mode and FL mode in ALA‐PDD group. The lesions of 225 patients visualized under the FL mode in the ALA‐PDD group diagnosed with non–muscle‐invasive bladder cancer were included in the analysis of diagnostic accuracy and the number of tumours or carcinoma in situ detected. Positive and negative lesions indicate those histologically classified as tumour‐positive and tumour‐negative, respectively. The number in parentheses below the statistical test result indicates the *p* value. Values of *p* < 0.05 were considered statistically significant.

Abbreviations: 5‐ALA, 5‐aminolevulinic acid; CI, confidence interval; CIS, carcinoma in situ; FL, fluorescent light; PDD, photodynamic diagnosis; Pts, patients; SD, standard deviation; WL, white light.

^a^ *p* values were calculated using McNemar test.

^b^ *p* values were calculated using Student's *t*‐test.

^c^ *p* values were calculated using Welch's *t*‐test.

### 3.3 Diagnostic accuracy by lesion in two 5‐ALA exposure time ranges under FL mode in ALA‐PDD group

The number of positive and negative lesions detected with each 5‐ALA exposure time was 295 and 569 in the 2–4 h group and 160 and 277 in the 4–8 h group, and the sensitivity under the FL mode in each case was 93.9% in the 2–4 h group and 91.3% in the 4–8 h group (Table 3A). There was no significant difference in sensitivity between the 5‐ALA exposure time ranges (*p* = 0.29). In addition, the specificity for each 5‐ALA exposure time was 74.7% for the 2–4 h group and 66.1% for the 4–8 h group, indicating a significant difference in specificity between the 5‐ALA exposure time ranges (*p* < 0.05), with a significantly lower specificity for the longer 5‐ALA exposure time range. On the other hand, the number of CIS positive lesions detected at each 5‐ALA exposure time was 83 in the 2–4 h group and 58 in the 4–8 h group, and the detection sensitivity of CIS positive lesions for each 5‐ALA exposure time under the FL mode in the ALA‐PDD group was 92.8% for the 2–4 h group and 93.1% for the 4–8 h group, indicating no significant difference in the sensitivity of detection of CIS positive lesions between the two exposure time ranges (*p* = 1.0) (Table 3B).

**TABLE 3:** Diagnostic accuracy by lesion in two 5‐ALA exposure time ranges under fluorescent light (FL) mode in ALA‐PDD group.

(A)						
5‐ALA exposure time (h)	No. of pts	FL(+)/total positive lesion	Sensitivity (%) (95% CI)	FL(−)/total negative lesion	Specificity (%) (95% CI)	Tumours/pts, mean ± *SD*
2–4	144	277/295	93.9 (90.4–96.2)	425/569	74.7 (70.9–78.2)	1.92 ± 1.25
4–8	67	146/160	91.3 (85.5–95.0)	183/277	66.1 (60.1–71.6)	2.18 ± 1.65
*p* value			0.29148^a^		*p* < 0.05^a^ (0.00882)	0.26312^b^

(B)				
5‐ALA exposure time (h)	No. of pts	FL(+)/CIS positive lesion	Sensitivity (%) (95% CI)	CIS/pts, mean ± *SD*
2–4	144	77/83	92.8 (84.4–97.0)	0.53 ± 1.15
4–8	67	54/58	93.1 (82.5–97.8)	0.81 ± 1.44
*p* value		1.000^c^		0.17782^b^

*Note*: (A) Comparison of diagnostic accuracy and number of tumours detected with two 5‐ALA exposure time ranges under FL mode; and (B) Comparison of diagnostic accuracy and number of carcinoma in situ detected for two 5‐ALA exposure time ranges under FL mode. Positive and negative lesions indicate those histologically classified as tumour‐positive and tumour‐negative, respectively. The number in parentheses below the statistical test result indicates the *p* value. Values of *p* < 0.05 were considered statistically significant.

Abbreviations: 5‐ALA, 5‐aminolevulinic acid; CI, confidence interval; CIS, carcinoma in situ; FL, fluorescent light; Pts, patients; *SD*, standard deviation; WL, white light.

^a^ *p* values were calculated using *χ* ^2^ test.

^b^ *p* values were calculated using Welch's *t*‐test.

^c^ *p* values were calculated using Fisher's exact test.

### 3.4 Analysis of number of patients with postoperative recurrence in WL and ALA‐PDD groups

**FIGURE 2:**
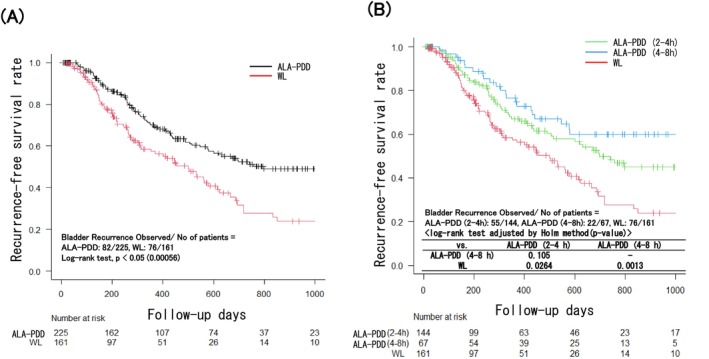
Comparison of recurrence free survival (RFS) between WL group and ALA‐PDD group. Comparison of RFS (A) between WL group and ALA‐PDD group (follow‐up ≤1000 days) and (B) between WL group and two 5‐ALA exposure time range groups in ALA PDD group (follow‐up ≤1000 days). Vertical marks on the plots represent censored events. ALA‐PDD, 5‐aminolevulinic acid hydrochloride photodynamic diagnosis; WL, white light.

Seventy‐six of 161 patients (47.2%) in the WL group and 82 of 225 patients (36.4%) in the ALA‐PDD group showed recurrence during follow‐up within 1000 days after initial TURBT (Figure 2). Kaplan–Meier curves showed that RFS was significantly longer in the ALA‐PDD group than in the WL group within 1000 days of the follow‐up period (*p* < 0.05). In addition, RFS was significantly longer in both the 2–4 h and 4–8 h groups of ALA‐PDD than in the WL group (*p* < 0.05). However, there was no significant difference in RFS between the 2–4 h and 4–8 h ALA‐PDD groups. The number of recurrences per 10 000 days determined using the person–time method was found to be 13.20/10 000 days in the WL group and 9.28/10000 days in the ALA‐PDD group (*p* < 0.05) (Table 4). Additionally, the recurrence frequencies per 10 000 days for the 5‐ALA exposure time 2–4 h group and the 4–8 h group were 10.43 and 6.43, respectively. Furthermore, the recurrence frequency was significantly lower only in the 4–8 h group compared with the WL group.

**TABLE 4:** Comparison of cumulative incidences of recurrence in NMIBC patients.

	WL	ALA‐PDD	*p* value	WL	5‐ALA exposure time (h)		*p* value
					2–4	4–8	
No. of patients	161	225^a^		161	144	67	
Cumulative incidence of bladder cancer recurrence	112	132		112	99	26	
Person‐days at risk	84 693	142 303		84 693	94 904	40 423	
Incidence in 10 000 days (95% CI)	13.2 (13.00–13.45)	9.28 (9.13–9.43)	*p* < 0.05^b^ (0.00550)	13.2 (13.00–13.45)	10.43 (10.24–10.63)	6.43 (6.19–6.68)	*p* < 0.05^b^ (0.00259)
Relative ratio for incidence (95% CI)	1	0.70 (0.54–0.90)		1	0.79 (0.60–1.03)	0.49 (0.32–0.75)	
Estimated days for bladder cancer recurrence	756	1078		756	984	1555	
Relative term for bladder cancer recurrence (95% CI)	1	1.43 (1.11–1.84)		1	1.27 (0.97–1.66)	2.06 (1.34–3.15)	
No. of patients with bladder cancer recurrence (%)	77 (47.8%)	87 (38.7%)	0.07265^b^	77 (47.8%)	60 (41.7%)	22 (32.8%)	0.10780^b^

*Note*: Cumulative incidence of recurrence was analysed by the person–time method. After confirming a significant difference (*p* < 0.05) between the three groups using the *χ* ^2^ test, multiple comparisons were performed using the *χ* ^2^ test with adjustments made by the Holm method, which demonstrated the following differences: WL versus 5‐ALA exposure time (4–8 h): *p* < 0.05 (0.003); WL versus 5‐ALA exposure time (2–4 h): *p* = 0.098; 5‐ALA exposure time (2–4 h) versus (4–8 h): *p* = 0.068. The number in parentheses below the statistical test result indicates the *p* value.

Abbreviations: ALA‐PDD, 5‐aminolevulinic acid hydrochloride photodynamic diagnosis; CI, confidence interval; NMIBC, non–muscle‐invasive bladder cancer; No., number; N.S., not significant; WL, white light.

^a^ The 225 patients in the ALA‐PDD group included patients with 5‐ALA exposure times of ‘less than 2 h’ and ‘exceeding 8 h’, which fall within the off‐label use range (see Figure 1).

^b^ *p* values were calculated using *χ* ^2^ test.

### 3.5 Comparison of PFS between WL and ALA‐PDD groups based on number of patients who exhibited disease progression

**FIGURE 3:**
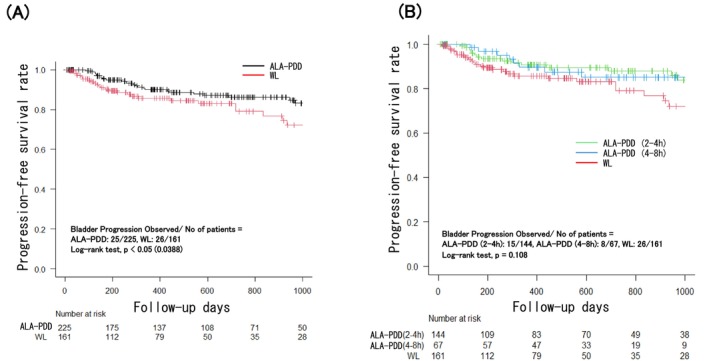
Comparison of progression free survival (PFS) between WL group and ALA‐PDD group. Comparison of PFS (A) between WL group and ALA‐PDD group (follow‐up ≤1000 days) and (B) between WL group and two 5‐ALA exposure time range groups in ALA‐PDD group (follow‐up ≤ 1000 days). Vertical marks on the plots represent censored events. ALA‐PDD, 5‐aminolevulinic acid hydrochloride photodynamic diagnosis; WL, white light.

Twenty‐six of 161 patients (16.1%) in the WL group and 25 of 225 patients (11.1%) in the ALA‐PDD group showed progression to MIBC within the 1000‐day follow‐up period after the initial TURBT (Figure 3). PFS was statistically longer in the ALA‐PDD group than in the WL group (*p* < 0.05). In addition, there was no significant difference in PFS among the two 5‐ALA exposure time groups and the WL group (*p* = 0.108).

## 4 DISCUSSION

We previously reported on the impact of ALA‐PDD on diagnostic accuracy and the effectiveness of treatment for NMIBC using real‐world data, as well as its impact on healthcare economics in the Japanese insurance system.^8^, ^10^, ^11^ In Japan, the appropriate utilization of 5‐ALA involved its oral administration 2 to 4 h before cystoscopy. Therefore, there has been no evidence of benefits of ALA‐PDD exceeding 4 h after 5‐ALA administration.

Taoka et al. reported that with extended 5‐ALA administration time (4–8 h before TURBT), ALA‐PDD under the FL mode exhibited significantly greater sensitivity in comparison to that under the WL mode (95.3% vs. 61.1%, *p* < 0.001), but lower specificity in comparison to observation under the WL mode (52.7% vs.. 95.2%, *p* < 0.001).^9^ With respect to AEs, of the 145 patients who received 5‐ALA, 136 (93.8%) and 75 (51.7%) experienced 377 AEs and 95 adverse reactions, respectively, most of which were Grade 1 or 2. No severe AEs were observed, and the tolerability profile was consistent with previously reported findings. Consequently, the authorized administration period for 5‐ALA in Japan has been changed to 2–8 h before TURBT, improving the convenience of ALA‐PDD for practical use.

In this study, the sensitivity of detection of positive lesions by FL mode during ALA‐PDD was 93.9% and 91.3% in the 2–4 h and 4–8 h 5‐ALA exposure groups, respectively, indicating no significant difference between two 5‐ALA exposure time groups (*p* = 0.34). This indicates that even if the time interval between the oral administration of 5‐ALA and insertion of the cystoscope is extended, the diagnostic sensitivity is comparable with that of the previously followed time period (2–4 h). On the other hand, the 2–4 h and 4–8 h groups under FL mode exhibited a specificity of 74.7% and 66.1%, respectively, indicating a significant difference between the two 5‐ALA exposure time groups (*p* < 0.05). Specificity with the FL mode during ALA‐PDD was found to be significantly lower with prolonged 5‐ALA exposure times. A clue to understanding this phenomenon may be found in the finding of Valdés et al., who reported a positive correlation between the amount of PpIX accumulating in brain tumour cells and cancer malignancy.^12^ Hence, our case may suggest that precancerous lesions have a lower PpIX production capacity than cancer cells, leading to a longer time for PpIX to reach a level that is visible by fluorescence. In addition, Matsuyama et al. reported that in ALA‐PDD, there were many false‐positive lesions that emitted red fluorescence but did not show malignant findings histopathologically, including lesions that harboured gene mutations that reflected precancerous conditions.^13^ Considering these reports, our results may suggest that as the 5‐ALA exposure time increases, the amount of PpIX accumulated in precancerous bladder lesions or in lesions with precancerous conditions involving genetic mutations increases to a level that can be detected by fluorescence, leading to a decrease in specificity due to the increase in false positives. The reduced specificity observed in the 4–8 h of 5‐ALA exposure group may have been due to detection of precancerous lesions or precancerous conditions with genetic mutations, rather than over‐detection of normal tissue. In 2025, National Cancer Center Japan, a research team of Ministry of Health, Labor and Welfare released ‘Monitoring of Cancer Incidence in Japan—Survival’, which presents survival rates by clinical stage and annual trends.^14^ The former shows the 5‐year net survival rates by stage of progression, including early stage cancer and distant metastasis, and highlights the importance of early detection. The latter shows that the 5‐year net survival rates for patients diagnosed with cancer between 2012 and 2015 have decreased compared with 1993–1996 (men: −10.6%, women: −5.9%), although the rates have increased for many cancers. Following this idea, the active removal of false positives in the 4–8 h 5‐ALA exposure group using ALA‐PDD technology might improve the survival rate of bladder cancer. Our hypothesis is that the reduced recurrence rate observed in the person–time method shown in Table 4 might have been due to the resection of precancerous lesions detected as false positives in the 4–8 h of 5‐ALA exposure group. However, further research is needed to evaluate the false positives with ALA‐PDD.

In this study, RFS duration was longer in the ALA‐PDD group than the WL group (*p* < 0.05) (Figure 2A). On the other hand, Figure 2B showed that there was a significant difference in RFS in both the 5‐ALA exposure time 2–4 h group and the 4–8 h group compared with the WL group (*p* < 0.05), but there was no significant difference in RFS between the two groups with different 5‐ALA exposure times (*p* = 0.105). It suggested similar treatment outcomes in terms of recurrence compared with the former conventional usage, suggesting that a better therapeutic effect is maintained for up to 8 h after 5‐ALA administration.

The number of recurrences per 10 000 days, as determined using the person–time method shown in Table 4, was 13.2, 10.43, and 6.43 in the WL group, 2–4 h group, and 4–8 h group of 5‐ALA exposure time, respectively. The difference between recurrence rates was significant only between the WL group and the 4–8 h group of 5‐ALA exposure time. This suggests that a favourable therapeutic outcome can be expected even if the 5‐ALA exposure time is set to 4–8 h in ALA‐PDD. However, there was no significant difference in PFS between 5‐ALA exposure times of 2–4 h and 4–8 h compared with the WL group. We estimated that the 5‐ALA exposure time had a minimal impact on disease progression. This is the first report on which ALA‐PDD has been demonstrated to result in statistically longer prolongation of PFS, though some reports have already demonstrated the significance of prolonged PFS with PDD‐TURBT compared with WL‐TURBT in large‐scale prospective studies and meta‐analyses of data from multi‐centre meta‐analysis integrated data.^15^ Notably, no report has demonstrated a significant PFS benefit of ALA‐PDD using real‐world data in a single‐centre retrospective analysis. We suppose that it is because a single‐centre study could eliminate variation between centres and enable highly accurate analysis, even with a small number of patients.

The present study has some limitations, such as its retrospective nature, small number of patients and insufficient follow‐up period, which make it difficult to draw firm conclusions. First, PFS and RFS in relation to 5‐ALA exposure time for ALA‐PDD should be analysed over a long‐term follow‐up period. Second, we did not perform a detailed analysis of AEs of ALA‐PDD, particularly in terms of hypotension. Therefore, the relationship between the extended follow‐up period and the incidence of 5‐ALA induced hypotension should also be examined in future studies.

In conclusion, consistent with our previous reports, this study demonstrated that the ALA‐PDD group exhibited higher sensitivity but lower specificity than the WL group, and that the former more effectively reduced bladder cancer recurrence compared with the latter. Furthermore, the clinical efficacy of ALA‐PDD, in terms of sensitivity and recurrence, was maintained even if the 5‐ALAadministration time was extended from 2–4 h to 2–8 h prior to TURBT.

## AUTHOR CONTRIBUTIONS

**Hideo Fukuhara:** Conceptualization; data curation; investigation; formal analysis; writing—original draft; writing—review and editing. **Ryu Shigehisa:** writing—review and editing. **Shinkuro Yamamoto:** writing—review and editing. **Satoshi Fukata:** writing—review and editing. **Kenta Saito:** formal analysis; writing—review and editing. **Yasuhiko Shibanaka:** formal analysis; writing—review and editing. **Keiji Inoue:** writing—review and editing.

## CONFLICT OF INTEREST STATEMENT

Kenta Saito and Yasuhiko Shibanaka are employees of SBI Pharmaceuticals Co., Ltd. No other authors in this manuscript have any conflicts of interest to disclose.

## Data Availability

The data supporting the findings of this study are available from the corresponding author upon reasonable request.
